# An increasing trend of rural infections of human influenza A (H7N9) from 2013 to 2017: A retrospective analysis of patient exposure histories in Zhejiang province, China

**DOI:** 10.1371/journal.pone.0193052

**Published:** 2018-02-15

**Authors:** Enfu Chen, Maggie H. Wang, Fan He, Riyang Sun, Wei Cheng, Benny C. Y. Zee, Steven Y. F. Lau, Xiaoxiao Wang, Ka Chun Chong

**Affiliations:** 1 Zhejiang Provincial Center for Disease Control and Prevention, Binjiang District, Hangzhou, Zhejiang, China; 2 School of Public Health and Primary Care, Faculty of Medicine, The Chinese University of Hong Kong, Hong Kong, China; 3 Clinical Trials and Biostatistics Laboratory, Shenzhen Research Institute, The Chinese University of Hong Kong, Shenzhen, China; Nanjing Agricultural University, CHINA

## Abstract

**Background:**

Although investigations have shown that closing live poultry markets (LPMs) is highly effective in controlling human influenza A (H7N9) infections, many of the urban LPMs were shut down, but rural LPMs remained open. This study aimed to compare the proportional changes between urban and rural infections in the Zhejiang province from 2013 to 2017 by analyzing the exposure histories of human cases.

**Methods:**

All laboratory-confirmed cases of H7N9 from 2013 (the first wave) to 2017 (the fifth wave) in the Zhejiang province of China were analyzed. Urban and rural infections were defined based on the locations of poultry exposure (direct and indirect) in urban areas (central towns) and rural areas (towns and villages on the outskirts of cities). A Chi-square trend test was used to compare the proportional trend between urban and rural infections over time and logistic regression was used to obtain the odds ratio by years.

**Results:**

From 2013 to 2017, a statistically significant trend in rural infections was observed (*p* <0.01). The incremental odds ratio by years of rural infections was 1.59 with 95% confidence intervals of 1.34 to 1.86. Each year, significant increases in the proportion of live poultry transactions in LPMS and poultry processing plants were detected in conjunction with an increased proportion of urban and rural infections.

**Conclusion:**

The empirical evidence indicated a need for heightened infection control measures in rural areas, such as serving rural farms and backyards as active surveillance points for the H7N9 virus. Other potential interventions such as the vaccination of poultry and extending the closure of LPMs to the provincial level require further careful investigations.

## Introduction

Since the first human influenza A/H7N9 infection was identified and confirmed by the Chinese government in March 2013, China has experienced five waves of human H7N9 influenza epidemics. By the end of the first wave in July 2013, a total of 135 confirmed cases, with 44 deaths, was reported [1]. The third epidemic wave, beginning in November 2014, included over 200 cases of H7N9 human infections [2]. In the fifth epidemic wave, the number of H7N9 cases was greater than that reported in earlier waves [3–5]. In addition, a greater number of western provinces was affected and a highly pathogenic form of the H7N9 influenza virus emerged [4]. In the Zhejiang province of China, 46 cases were identified, including 11 deaths related to the initial H7N9 epidemic of 2013 [6]. In 2014, the number of laboratory-confirmed cases gradually increased to 93, of which 39 resulted in fatalities [7, 8]. At the end of the third epidemic wave, there were 185 confirmed cases, resulting in 71 deaths fatalities [9]. Of all the Chinese provinces, Zhejiang was the region with the most confirmed cases of infection.

Numerous studies have shown that exposure to poultry in live poultry markets (LPMs) is the most common and probable transmission route of H7N9 infections in humans [10–12]. Thus, closing LPMs has been proven to be an effective control measure in the prevention of zoonotic transmissions [13–15]. In the Zhejiang province, approximately 30% of LPMs in central towns were shut down from April 13 to April 22, 2013, following the advice of the Zhejiang Center for Disease Control (CDC). After the LPM closures, the number of infections dropped substantially, with the number of human infections dropping by 92% in Hangzhou City, Zhejiang [9, 14, 15]. In the second epidemic wave, approximately 71% of LPMs were closed from January 1 to February 15, 2014. Similar significant decreases were observed in other cities, with the mean number of daily infections decreasing by 99% in Shanghai and by 97% in Nanjing [15]. Given the success of incidence reduction from LPM closures, the Zhejiang CDC advised the shutdown of all LPMs in urban towns from the third epidemic wave onwards.

However, the current infection control strategy of LPM closures may not be the best method of disease management, especially for the fifth epidemic wave, in which the epidemic size increased after LPM closures. Many of the LPMs in urban central regions were shut down, but LPMs in rural regions remained opened and a relatively proportional increase in the number of H7N9-infected towns and H7N9 cases was observed. In 2014, there was a reported case of a re-emergent H7N9 infection in the Zhejiang province despite the closure of most LPMs in the central towns. In this case, the infection was transmitted by an individual who had visited the LPMs in the rural Xinren village [16]. The proportion of positive environmental specimens from LPMs was also significantly higher in rural areas than that in urban markets [17]. Therefore, this study aimed to determine the difference in proportional changes between urban and rural infections in the Zhejiang province from 2013 to 2017 by analyzing the exposure histories of cases. The majority of the human infection sources was expected to change from LPMs to rural reservoirs, such as poultry farms and backyards, possibly as a result of limited control measures in rural settings or limited alternative routes for poultry trading.

## Methods

### Data collection

This study analyzed all identified human cases of influenza A (H7N9) (n = 306) from 2013 to 2017 in Zhejiang, an eastern province in China with 11 prefectures and an approximate population of 56 million. The study period (reported date of the first case to the last case) spans five waves of H7N9 epidemics from April 3 to April 27, 2013 (W1), from October 15, 2013 to June 16, 2014 (W2), from November 26, 2014 to June 2, 2015 (W3), from September 26, 2015 to June 29, 2016 (W4), and from October 8, 2016 to June 12, 2017 (W5). The last updated date for reported cases was July 12, 2017.

Laboratory-confirmed cases were identified according to the protocol from the National Health and Family Planning Commission of the People’s Republic of China, and the procedure of laboratory tests has been previously described [18, 19]. All notifiable cases have to be recorded in the China Information System for Disease Control and Prevention by all hospitals in Zhejiang. Data on the following factors were collected for each patient via face-to-face interviews: demographics, date of illness onset and hospitalization, details of exposure histories, such as previous visits to LPMs or family farms, and poultry contact.

### Exposure Information

Urban infections and rural infections were defined based on the location of where the patient had direct or indirect contact with poultry. According to the official definition [20], an urban location was defined as a settlement with a non-agricultural population, including cities and central towns, that either had a population density >1500 inhabitants per square kilometer or had major municipal government facilities. A rural location was defined as a settlement with an agricultural population dominated by agricultural production. In China, rural areas were commonly regarded as settlements outside urban areas, such as towns and villages on the outskirts of cities. Subjects without clear locations, for example, a patient having contact with chickens in a bus, were excluded from the analysis. The period of a patient’s exposure history was limited to two weeks before illness onset.

The types of exposure were categorized into the following: transactions (selling or buying) poultry in LPMs, visiting LPMs, slaughtering or processing poultry in plants, raising poultry in farms or backyards, and multiple exposures in rural and urban places.

### Statistical analysis

The primary endpoint of this study was the proportional trend of rural infections over time, which was analyzed using the Chi-square trend test. Univariate incremental odds ratio (ORR) by time (order of waves) and the ORR adjusted for age and gender were obtained by logistic regressions. In addition, the 95% confidence intervals (CI) were obtained. Descriptive statistics were used to evaluate the demographics, occupations, and exposure types. The trends of variables by time, such as occupation and exposure history, were also tested using the Chi-square trend test. To assess the changes of the association (between variables and rural infections) by time, logistic regression was used to obtain the *p*-value of the interaction term between the variable and time. *P*-values<0.05 were considered to be statistically significant. All analyses were conducted using SAS 9.4 software.

### Ethical considerations

This study was reviewed and approved by the Medical Ethics Committee of the Zhejiang CDC. Informed consents were exempt from the ethics committee in accordance to the CDC policy of continuing public health investigations of notifiable infectious diseases. The data were analysed anonymously and are provided in S1 File.

## Results

**Fig 1:**
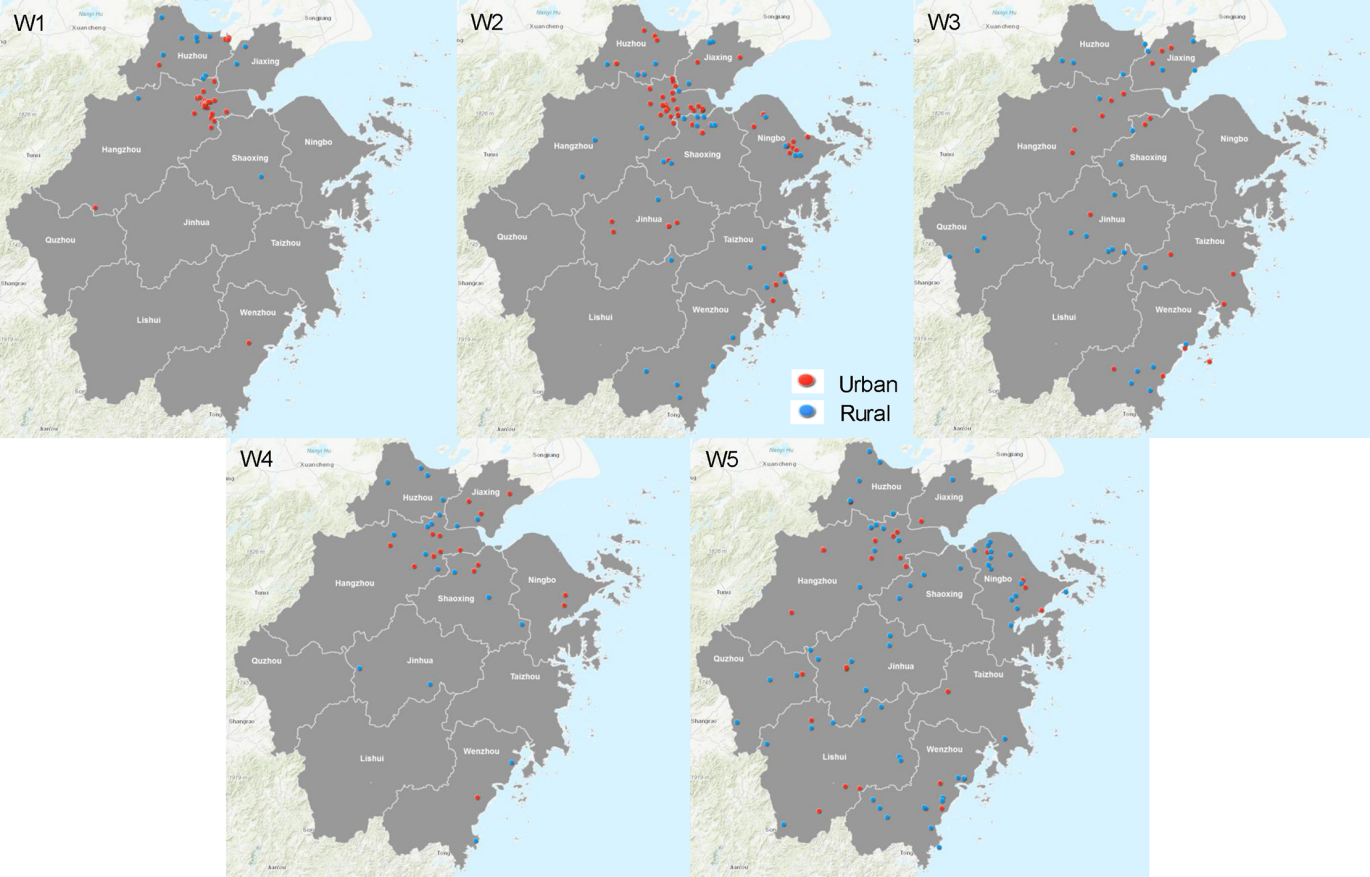
Spatial distribution of urban (red) and rural (blue) H7N9 infections from the first to the fifth epidemic waves (W1 to W5) from April 2013 to June 2017 in the Zhejiang province, China. W1: First wave (April 3—April 27, 2013); W2: Second wave (October 15, 2013—June 16, 2014); W3: Third wave (November 26, 2014—June 2, 2015); W4: Forth wave (September 26, 2015—June 29, 2016); W5: Fifth wave (October 8, 2016—June 12, 2017).

According to the clinical data, spatial distribution between the urban and rural cases changed from the first to fifth epidemic wave (Fig 1). As illustrated, most of the reported urban cases in the first wave were around the central city of Hangzhou, while most of the rural cases were located in the northern part of Zhejiang. The distribution of cases became more disperse in the second and third waves, with the location of the cases found to be almost entirely sequestered to the outskirts of the province in the fifth wave. No obvious heterogeneity in the spatial distributions of cases was observed between genders (S1 Fig) and between age groups (S2 Fig).

**Fig 2:**
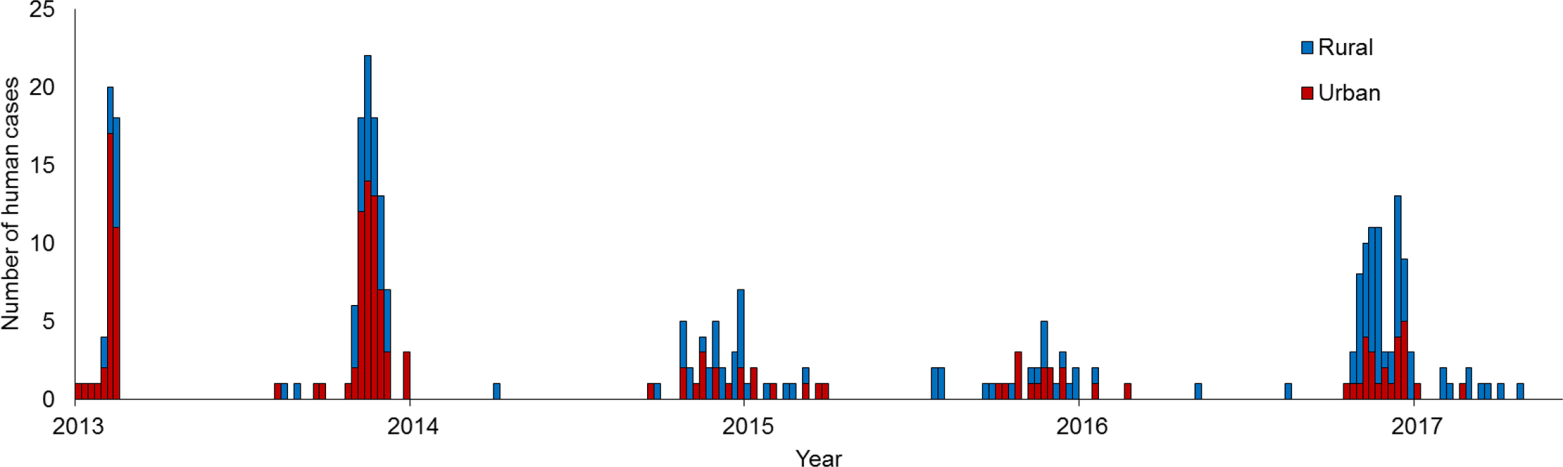
Temporal distribution of urban (red) and rural (blue) H7N9 infections in the five epidemic saves from April 2013 to June 2017 in the Zhejiang province, China.

The temporal distribution of human H7N9 cases is presented in Fig 2. In the study period from 2013 to 2017 (W1 to W5), 26% (12/46), 38% (36/94), 60% (27/45), 56% (19/34), and 71% (62/87) rural infections were reported in the Zhejiang province, respectively. A statistically significant trend was observed in the number of rural infections from W1 to W5 (*p*<0.001) and the incremental ORR over time was 1.59 (95% CI: 1.34 to 1.86), meaning the odds of rural infections increased by approximately 59% each epidemic year. After adjusting for age and gender, the incremental ORR was 1.63 (95% CI: 1.37 to 1.93).

Table 1 summarizes the demographics and epidemiological characteristics of the urban and rural cases. Overall, the median age for both urban and rural infections as between 54- to 65-years-old. The majority of the patients (>80%) were adults aged greater than 35 years, with approximately 50% being over the age of 60. Among the urban infections, the male proportion significantly decreased from 68% (23/34) in W1 to 64% (37/58) in W2, to 44% (8/18) in W3, to 33% (5/15) in W4, and to 52% (13/25) in W5. No statistically significant change in the association between urban and rural infections and occupations was detected. The majority of the rural patients were farmers (>40%), while the majority of the urban patients were homemakers (>30%). In 2014, an increase in the proportion of rural patients who worked in LPMs was observed (*p* <0.01); however, this proportion later decreased to zero. The mortality proportions were similar between rural and urban infections.

**Table 1:** Demographics and epidemiological characteristics of rural and urban infections of H7N9 from 2013 to 2017 (W1 to W5).

	W1		W2		W3		W4		W5		P-value of trends^a^		P-value of interaction^b^
Variables	Urban (n = 34)	Rural (n = 12)	Urban (n = 58)	Rural (n = 36)	Urban (n = 18)	Rural (n = 27)	Urban (n = 15)	Rural (n = 19)	Urban (n = 25)	Rural (n = 62)	Urban	Rural	
Male	23 (68%)	6 (50%)	37 (64%)	27 (75%)	8 (44%)	22 (82%)	5 (33%)	14 (74%)	13 (52%)	40 (65%)	0.04	0.71	0.18
Age (median, IQR)	63, 50–73	55, 43–66	58, 43–67	61, 47–67	60, 45–68	56, 49–65	54, 49–58	65, 53–70	60, 46–73	60, 51–69	0.90	0.22	0.58
Age<18	0	0	3 (5%)	0	0	0	1 (7%)	0	0	0			
Age 18–35	1 (3%)	1 (8%)	7 (12%)	4 (11%)	3 (17%)	2 (7%)	1 (7%)	0	0	7 (11%)			
Age 36–60	13 (38%)	7 (58%)	24 (41%)	14 (39%)	7 (39%)	14 (52%)	10 (67%)	8 (42%)	13 (52%)	25 (40%)			
Age>60	20 (59%)	4 (33%)	24 (41%)	18 (50%)	8 (44%)	11 (41%)	3 (20%)	11 (58%)	12 (48%)	30 (48%)			
Occupation													
Farmer	8 (24%)	8 (67%)	19 (33%)	22 (61%)	5 (28%)	12 (44%)	0	11 (58%)	7 (28%)	38 (61%)	0.49	0.95	0.65
Homemaker	17 (50%)	1 (8%)	20 (34%)	5 (14%)	9 (50%)	4 (15%)	6 (40%)	1 (5%)	10 (40%)	10 (16%)	0.74	0.64	0.70
Work in LPMs	0	0	8 (14%)	10 (28%)	1 (6%)	2 (7%)	2 (13%)	0	0	0	0.73	<0.01	0.09
Others	9 (26%)	3 (25%)	15 (26%)	5 (14%)	3 (17%)	10 (37%)	8 (53%)	7 (37%)	8 (32%)	14 (23%)	0.27	0.67	0.74
Dead	9 (26%)	2 (17%)	27 (47%)	12 (33%)	7 (39%)	17 (63%)	5 (33%)	8 (42%)	8 (32%)	21 (34%)	0.88	0.81	0.29
Exposure history													
Transactions (selling or buying) poultry in LPMs	6 (18%)	0	16 (28%)	13 (36%)	7 (39%)	8 (30%)	8 (53%)	4 (21%)	9 (36%)	28 (45%)	0.03	0.02	0.77
Visiting LPMs	20 (59%)	4 (33%)	37 (64%)	22 (61%)	11 (61%)	16 (59%)	9 (60%)	8 (42%)	19 (76%)	44 (71%)	0.67	0.70	0.86
Slaughtering or processing poultry in plants	0	0	14 (24%)	9 (25%)	1 (6%)	4 (15%)	7 (47%)	6 (32%)	9 (36%)	24 (39%)	<0.01	<0.01	0.77
Rearing poultry in farms or backyards	19 (56%)	7 (58%)	17 (29%)	12 (33%)	8 (44%)	16 (59%)	1 (7%)	15 (79%)	10 (40%)	33 (53%)	0.15	0.22	0.11

IQR: Inter-quantile range; W1: First wave (April 3—April 27, 2013); W2: Second wave (October 15, 2013—June 16, 2014); W3: Third wave (November 26, 2014—June 2, 2015); W4: Forth wave (September 26, 2015—June 29, 2016); W5: Fifth wave (October 8, 2016—June 12, 2017)

^a^P-values were obtained by testing the variable over time using Chi-square trend test.

^b^P-values were obtained by testing the interaction term of the variable and time (order of epidemics) in logistic regressions

The analysis of the exposure histories of patients indicated that there were significant increases in the proportions of transactions (selling or buying) of live poultry in LPMs in both urban and rural infections. The proportion of urban infections increased each year from 18% in 2013 to 53% in 2016, followed by a dip to 36% in 2017 (*p* = 0.03). The proportion of rural infections increased from 0% in 2013 to 36% in 2014 and decreased to 30% and 21% in 2015 and 2016, respectively; however, the highest proportion (45%) was reached in 2017 (*p* = 0.02). The number of cases resulting from exposure to slaughtering or processing poultry increased significantly by year (*p* <0.01), from 0% in 2013 to 24%, 6%, 47%, and 36% in 2014 to 2017 for urban cases, respectively, and from 0% in 2013 to 25%, 15%, 32%, and 39% in 2014 to 2017 for rural cases, respectively. Among the rural cases, poultry rearing was one of the major sources of rural infection, maintaining an infection proportion more than 30% infection cases in every year (Table 1).

## Discussion

In this study, the changes in proportions between urban and rural infections of the human influenza A (H7N9) in the Zhejiang province from 2013 to 2017, spanning a total of five epidemic waves, were analyzed. Through the analysis of case exposure histories, this study found significant increase in the proportions of rural infections over time, even though there were drops in overall incidence. This finding is believed to be the result of insufficient infection control measures in rural areas, for example, a lack of surveillance for poultry rearing in rural towns. This study provides empirical evidence for the relative increases in human H7N9 cases observed in rural regions since the start of the H7N9 epidemics. A previous study indicated that highly pathogenic H7N9 infections that emerged during the fifth epidemic were likely associated with exposures to infected or dead poultry in rural areas [21]. The increase in proportion of rural infections may also be the result of residents traveling to rural or semi-rural areas to purchase poultry after the closure of urban LPMs, especially during the period of growing integration of urban and rural towns in China [22]. In addition, this study found significant increases in the proportions of live poultry transactions in LPMs and the processing of poultry in plants in both urban and rural infections over the years. Specifically, numbers of rural cases steadily increased. This finding indicated that direct poultry contact remained a potential source of infection, especially in rural areas. A previous survey in Zhejiang revealed that residents did not decrease or stop eating poultry, even though more than half of them expressed concern about contracting the H7N9 virus [23]. Similarly, other studies indicated that a low perception of severity and acceptance of preventive measures among the public were likely associated with the risk of H7N9 infections [24–26].

In addition to the LPM exposure, this study found that poultry rearing in farms or backyards was another major source of exposure, especially in the rural cases, in which raising poultry in backyards is very common to rural homemakers in China. A previous survey also indicated that more than half of the rural households had a history of backyard poultry breeding [27]. The risk of rural infections increases with such frequent exposure to a contaminated environment.

The results of this study are in accordance with a recent study conducted by He et al. [17], in which an increasing rate in the prevalence of the H7N9 virus was observed in environment samples of rural towns. He at al. found that when compared with the initial epidemic, the proportions of positive samples in the third epidemic increased approximately six-fold in the third wave. With a high correlation between human infections and positive environment samples [28], larger human H7N9 outbreaks are likely to occur in rural areas in the future.

Based on the findings of the present study, more infection control measures should be implemented in rural areas. Although LPM closures were unable to control the transmission and proliferation of the fifth epidemic in Zhejiang, extending market closures to the provincial level or even banning the presence of poultry around homes could be considered as one of the mitigation measures. Since 2006, the Hong Kong government has banned the keeping of backyard poultry and has achieved a success in minimizing the risk of a local avian influenza outbreak [29]. Yet, in Hong Kong, keeping poultry around the home was not common, as there are few rural areas in Hong Kong. Conversely, this mandate would be challenging to implement in rural towns of China because rearing poultry in the backyard is common amongst many households in these regions. With a great demand of live poultry in China, especially during festivals, such as the Chinese New Year, which that traditionally use live chickens for celebrations, even a temporary prohibition of poultry trade would raise economic and social concerns [23]. Therefore, cost-effectiveness analysis for different scales of banning poultry trade or backyard rearing is warranted. Multi-sector collaboration is also recommended to register households having live poultry, as these places should serve as active surveillance points for the H7N9 virus.

While prevention measures, such as species segregation, centralized environment disinfection, and annual rest days, are difficult to impose on rural poultry farms and backyards, vaccination of domestic poultry could be a potential alternative that would reduce the susceptibility to and probability of zoonotic transmissions [30]. Vaccinations would not only save the poultry from culling, it would also prevent income losses of farmers and live poultry workers caused by LPM closures or poultry keeping bans. A recent investigation showed that a newly developed vaccine was able to induce higher immunity to H7N9 strain in chickens [31]. Recently, the Ministry of Agriculture of China conducted the first H7N9 immunization campaign targeting species, including broiler chickens, egg-laying hens, geese, and ducks, in southern China, which reported more than 300 human cases during the 2016–2017 H7N9 outbreak [32]. However, the use of mass poultry vaccination for avian influenza control remains controversial [5, 30, 33]. Large-scale immunizations for the prevention of avian influenza A/H5N1 infections among poultry have been conducted in China since 2004 [5, 34], but vaccination failures were successively reported, probably as a result of antigenic drifts and immune escapes [35]. Post-vaccination monitoring programs with a high level of coverage are thus needed in the future.

The present study had several major limitations. The analysis indicated a significant increase in the proportion of rural infections over the years, but the study could not confirm whether or not the relationship resulted from a shift of transmissions from urban areas to rural areas via poultry trade and transportation because of there was a lack of relevant trade network data. Further, patients who did not have any exposure history to poultry were excluded from the analysis because they could have potentially been infected by human-to-human transmission [3, 9, 36, 37]. Although there is limited evidence on the human-to-human infection pathway and the main transmission route remains associated with LPM exposure, some studies have shown that ferrets can be a mammalian model for H7N9 transmission, spreading the virus via airborne routes and direct-contact with other animals, which suggests the possibility of viral evolution for human-to-human transmission [4, 38]. In addition, as with all cases, the exposure histories of the patients could be subjected to recall bias. Another major limitation of the present study is the under-reporting in surveillance, as most H7N9 cases could be asymptomatic or present mild conditions. With limited health care resources in rural areas, infections may not be successfully identified.

In conclusion, the findings of the study support the need for more infection control measures in rural areas, such as serving rural farms and backyards as active surveillance points for the H7N9 virus. Other potential interventions such as the vaccination of poultry and extending the closure of LPMs to the provincial level require further careful investigation.

## Supporting information

S1 Fig Spatial distributions of cases by genders. (PDF)

S2 Fig Spatial distributions of cases by age groups. (PDF)

S1 File Study data in SPSS format. (SAV)
