# Supplementary figures and images for: An increasing trend of rural infections of human influenza A (H7N9) from 2013 to 2017: A retrospective analysis of patient exposure histories in Zhejiang province, China
Source: PLoS One. 2018 Feb 15;13(2):e0193052. doi: 10.1371/journal.pone.0193052 (PMC5814046; doi:10.1371/journal.pone.0193052)

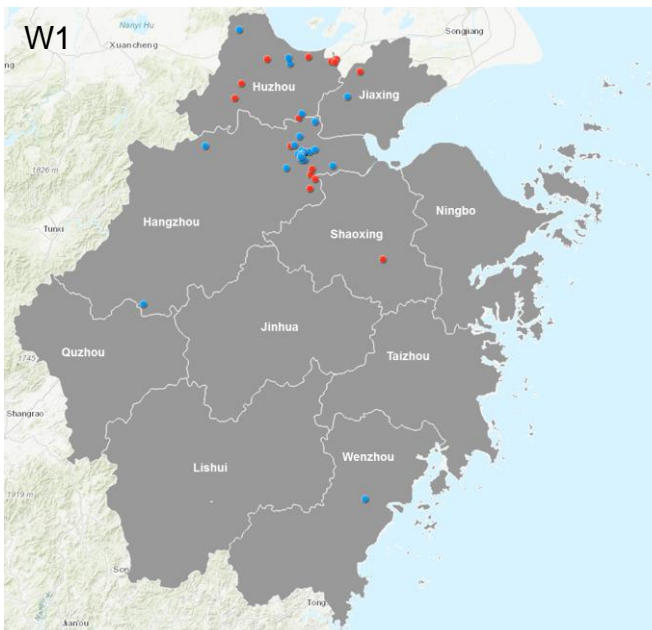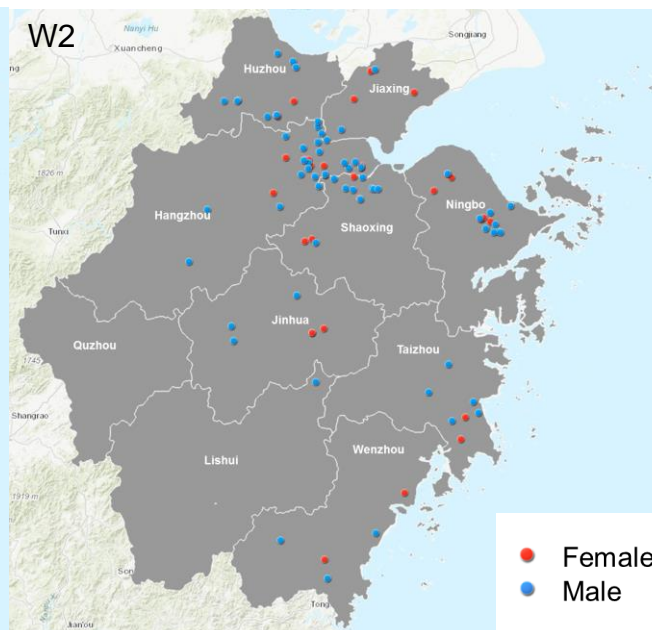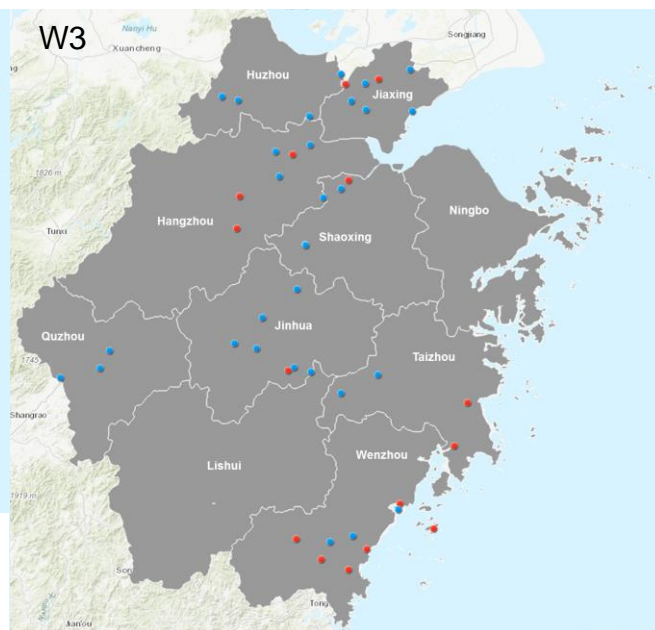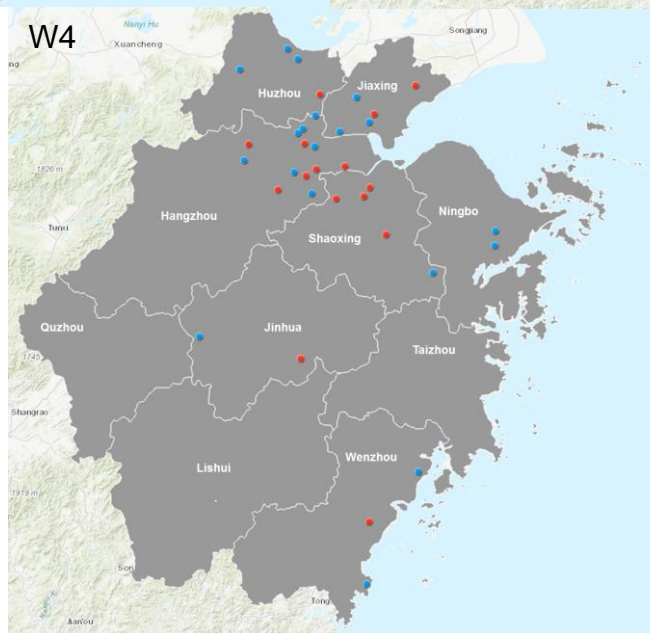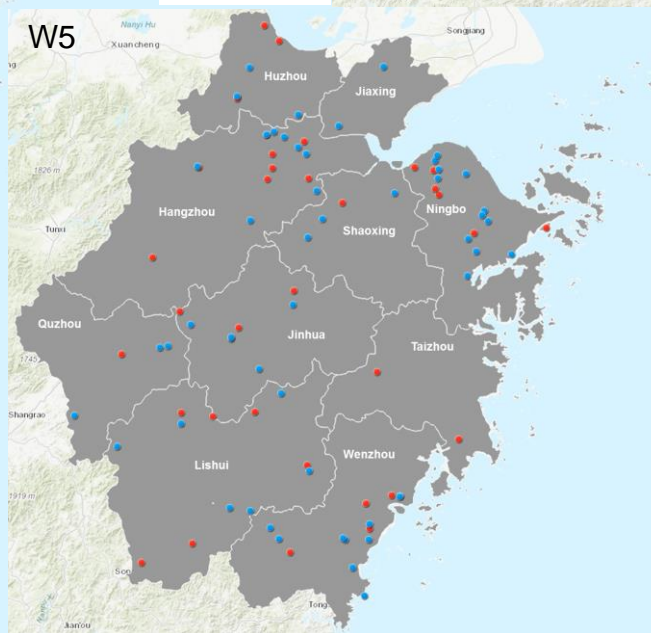

Supplement: S1 Fig — (PDF) [file pone.0193052.s001.pdf]

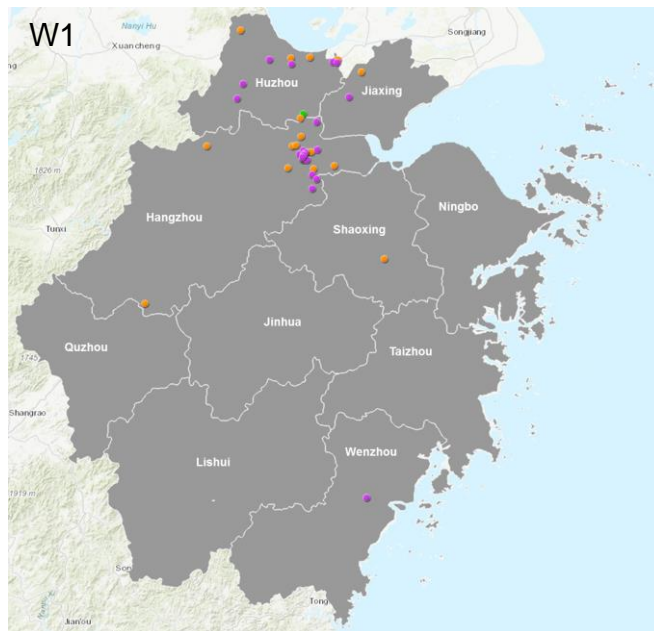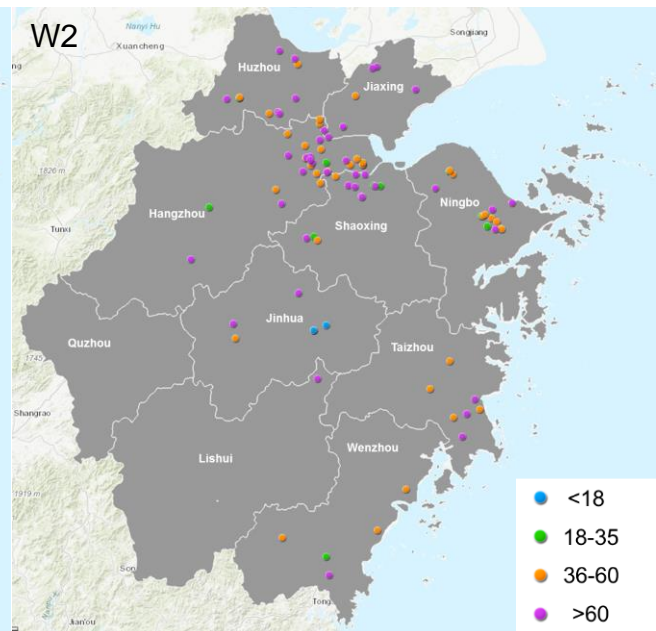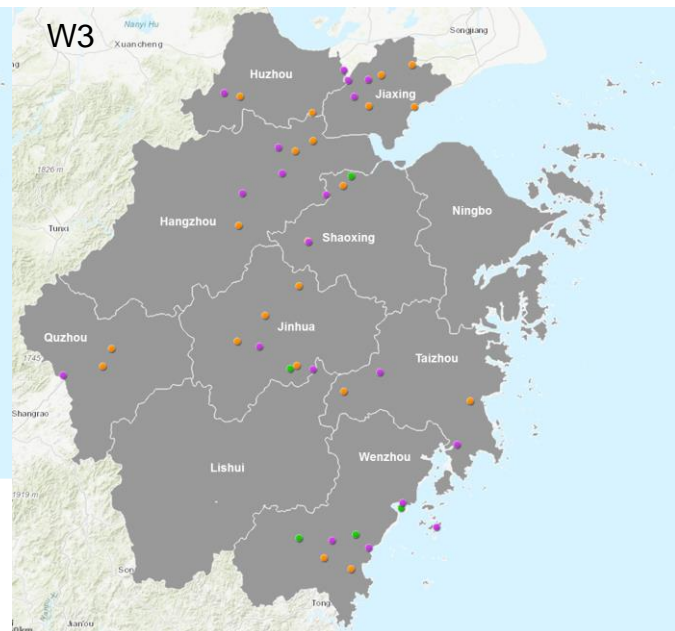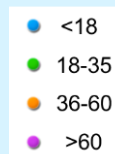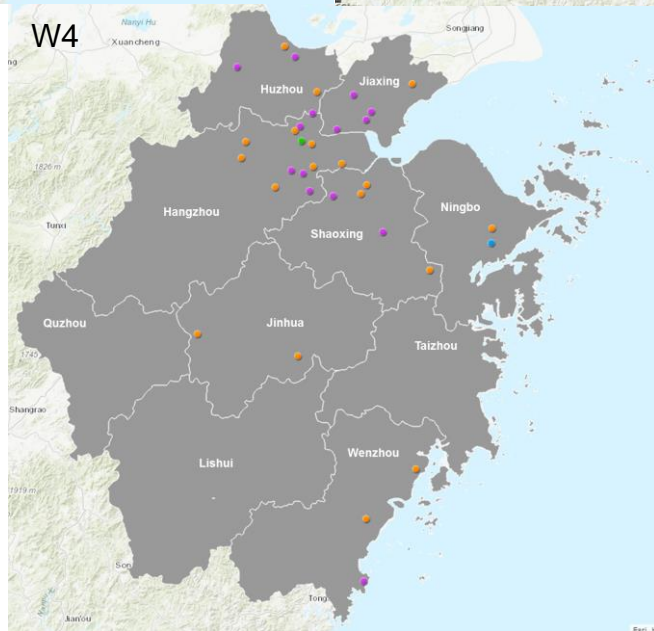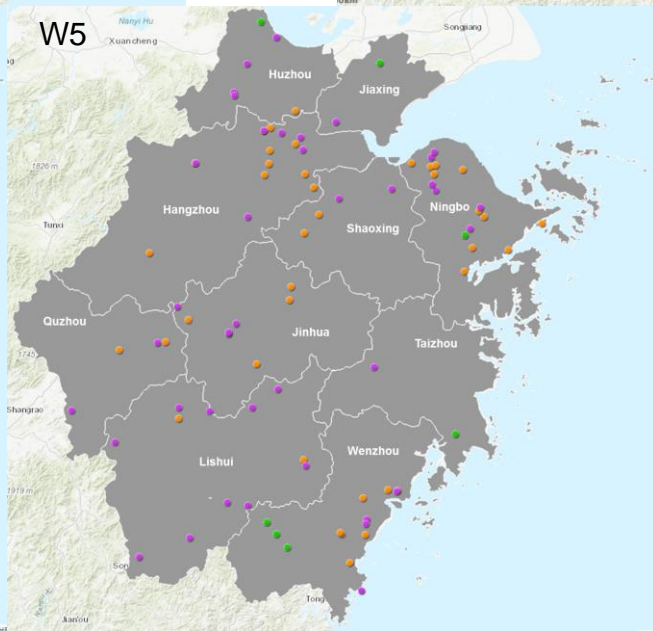

Supplement: S2 Fig — (PDF) [file pone.0193052.s002.pdf]
